# Overall Dietary Quality Relates to Gut Microbiota Diversity and Abundance

**DOI:** 10.3390/ijms20081835

**Published:** 2019-04-13

**Authors:** Kirsi Laitinen, Kati Mokkala

**Affiliations:** Institute of Biomedicine, University of Turku, FI-20014 Turun yliopisto, 20500 Turku, Finland; kamamo@utu.fi

**Keywords:** diet, dietary quality, gut microbiota, microbial diversity, whole grain, vegetables

## Abstract

Disturbances in gut microbiota homeostasis may have metabolic consequences with potentially serious clinical manifestations. Diet influences the host’s metabolic health in several ways, either directly or indirectly by modulating the composition and function of gut microbiota. This study investigated the extent to which dietary quality is reflected in gut microbiota diversity in overweight and obese pregnant women at risk for metabolic complications. Dietary quality was measured by a validated index of diet quality (IDQ) and microbiota composition was analyzed using 16SrRNA gene sequencing from 84 women pregnant less than 18 weeks. The alpha diversity, measured as Chao1, observed operational taxonomic units (OTUs), phylogenetic diversity, and the Shannon index were calculated. The IDQ score correlated positively with the Shannon index (rho = 0.319, *p* = 0.003), but not with the other indexes. The women who had the highest dietary quality (highest IDQ quartile) had higher gut microbiota diversity in all the investigated indexes, when compared to the women with the lowest dietary quality (lowest IDQ quartile; *p* < 0.032). Consequently, a higher dietary quality was reflected in a higher gut microbiota diversity. The presented approach may aid in devising new tools for dietary counseling aiming at holistic health, as well as in microbiome studies, to control for dietary variance.

## 1. Introduction

Disturbances in gut microbiota homeostasis may have metabolic consequences with potentially serious clinical manifestations. Diet can influence metabolic health of the host in several ways, either directly or by modulating the composition and function of the gut microbiota. This may be of particular importance during pregnancy as both diet and gut microbiota may contribute to the health of pregnant women and their offspring, as demonstrated by the presence of microbial disturbances in subjects with gestational diabetes [1,2,3].

Individual nutrients have essential and beneficial properties in maintaining and in promoting human health. Good examples are the deficiency disorders resulting from a lack of vitamins [4]. Ultimately it is the combination of foods and the diet as a whole which are most likely to account for a diet’s health-advancing properties (e.g., not only with regard to noncommunicable lifestyle related conditions, such as diabetes and cardiovascular disease, but also with respect to pregnancy-related conditions) [5,6,7]. Dietary index scores have been used for estimating the overall quality of a diet (i.e., food choices that comply with the recommended nutrient intakes) [8,9]. The general depicters of a healthy dietary pattern include high intakes of fruits and vegetables, high-fiber whole grain products, fish, the selection of low-fat dairy, meats, and low-sugar foods. Interestingly, the same healthy dietary patterns have been related to the composition of the gut microbiota [10]. Evidence from groups of individuals consuming diets from geographically or culturally differing areas, like vegetarian diets as compared to omnivore diets [11] or African diets compared to Western diets [12,13], have detected differences in gut microbiota composition. It is less well known whether more subtle changes in the diet (i.e., from defined groups of individuals who are choosing different foods when composing their diet), are reflected in their gut microbiota composition. Experimental evidence indicated that this is the case [14]. When considering microbiota, a high diversity has been generally considered as beneficial for health [15]. Lowered gut microbiota diversity has been observed in association with metabolic aberrations, including low-grade inflammation, insulin resistance, and dyslipidemia [16,17,18]. The mechanisms whereby diverse gut microbiota may benefit health include increased production of metabolites like anti-inflammatory short chain fatty acids and decreased production of inflammatory mediators, such as lipopolysaccharide [15].

The aim of this study was to investigate whether dietary quality measured with a validated index of diet quality (IDQ) would be reflected in gut microbiota diversity, analyzed using 16S rRNA gene sequencing, in overweight and obese pregnant women, a high-risk group for developing metabolic complications. It is demonstrated here that a higher dietary quality was related to a higher gut microbiota diversity. The key components of the IDQ that were related to microbiota diversity were daily consumption of whole grains and vegetables. Furthermore, a high IDQ was related to particular microbial abundances, primarily to the genus *Coprococcus* and species *Faecalibacterium prausnitzii,* as well as an unknown species in the family *Barnciellaceae*, whereas an unknown species of the genus *Sutterella* was related to a lower dietary quality.

## 2. Results

### 2.1. The Study Population

The mean age of the participating women was 30 years, almost half were obese, the mean pre-pregnancy body mass index (BMI) being 30.3, one out of every three was expecting her first child, and more than half (58%) were highly educated with a college or university degree (Table 1). The dietary quality of the women was evaluated in early pregnancy; the quality of the diet estimated to be good in 49% and poor in 51% of the women, using the categorization set in the previous IDQ validation report [9].

### 2.2. Dietary Quality in Relation to Gut Microbiota Diversity

**Figure 1:**
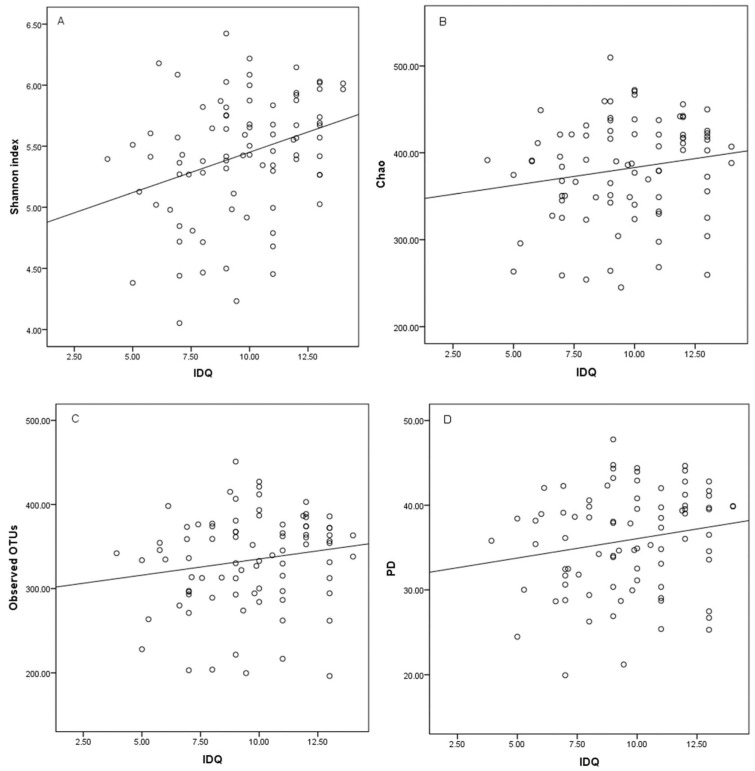
Correlations between index of diet quality (IDQ) score and gut microbiota diversity indexes: (**a**) Shannon index, (**b**) Chao index, (**c**) observed operational taxonomic units (OTUs), (**d**) phylogenetic diversity (PD) (*n* = 84).

A higher dietary quality was associated with a higher gut microbiota diversity. This was attributable to the positive correlation between the IDQ score and the Shannon index value, the diversity index that in addition to the presence of taxa, considers the number of times that each taxon is observed (rho = 0.319, *p* = 0.003) (Figure 1a). No relationship between IDQ score and microbiota diversity was detected when the diversity indexes only considered the presence of taxa (i.e., at phylogenetic, operational taxonomic units (OTUs), or species abundance levels): phylogenetic diversity (PD) (rho = 0.195, *p* = 0.075), observed OTUs (rho = 0.176, *p* = 0.110), or Chao (rho = 0.174, *p* = 0.114) indices (Figure 1b–d). Instead, when the extremities of the IDQ score (i.e., the individuals best adhering to the dietary recommendations were compared to those least adhering), were evaluated, a clear relationship was evident with all of the investigated gut microbiota indexes (*p* < 0.032); as compared to those in women in the lowest IDQ quartile, the women who had the highest IDQ scores (i.e., who were in the top IDQ quartile), had the highest gut microbiota diversity index values (Table 2). Furthermore, higher Shannon index values, but not the other microbiota diversity indexes, were detected in women with good dietary quality (i.e., categorized IDQ score) when compared to those with a poor dietary quality (Table 3).

As the dietary quality was best related to the Shannon microbiota diversity index, we further evaluated the role of the individual IDQ food groups as determinants of the Shannon index. The number of days in a week that whole grains (rho = 0.264, *p* = 0.015) and vegetables (rho = 0.265, *p* = 0.015) were consumed correlated with higher Shannon index values. Instead, consumption of dairy products, fruits and berries or fruit juices, fish, sugar-containing soft drinks or sweets, and chocolates was not correlated with the Shannon index values. Furthermore, daily consumption (seven days/week compared to six or fewer days per week) of whole grains and vegetables correlated with higher Shannon index values (Table 4). Most of the women, 58%, consumed whole grains every day, and 67% ate vegetables on a daily basis.

### 2.3. Dietary Quality in Relation to Gut Microbiota Abundancies

**Figure 2:**
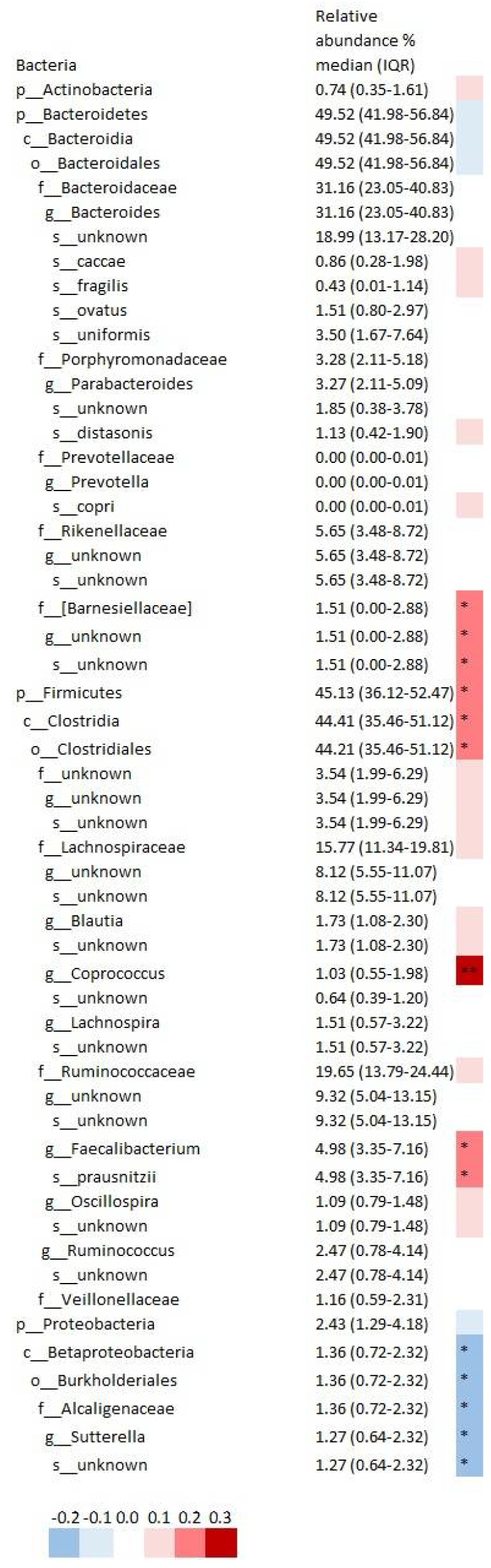
Correlations between IDQ scores and the relative abundance of bacteria. * FDR < 0.2, ** FDR < 0.1. The *p*-values for the correlation were corrected for multiple testing. The false discovery rate (FDR) was 0.2 *, but also those with FDR < 0.1 are shown **. The colors represent the Spearman’s rho (blue: negative correlation, red: positive correlation).

**Figure 3:**
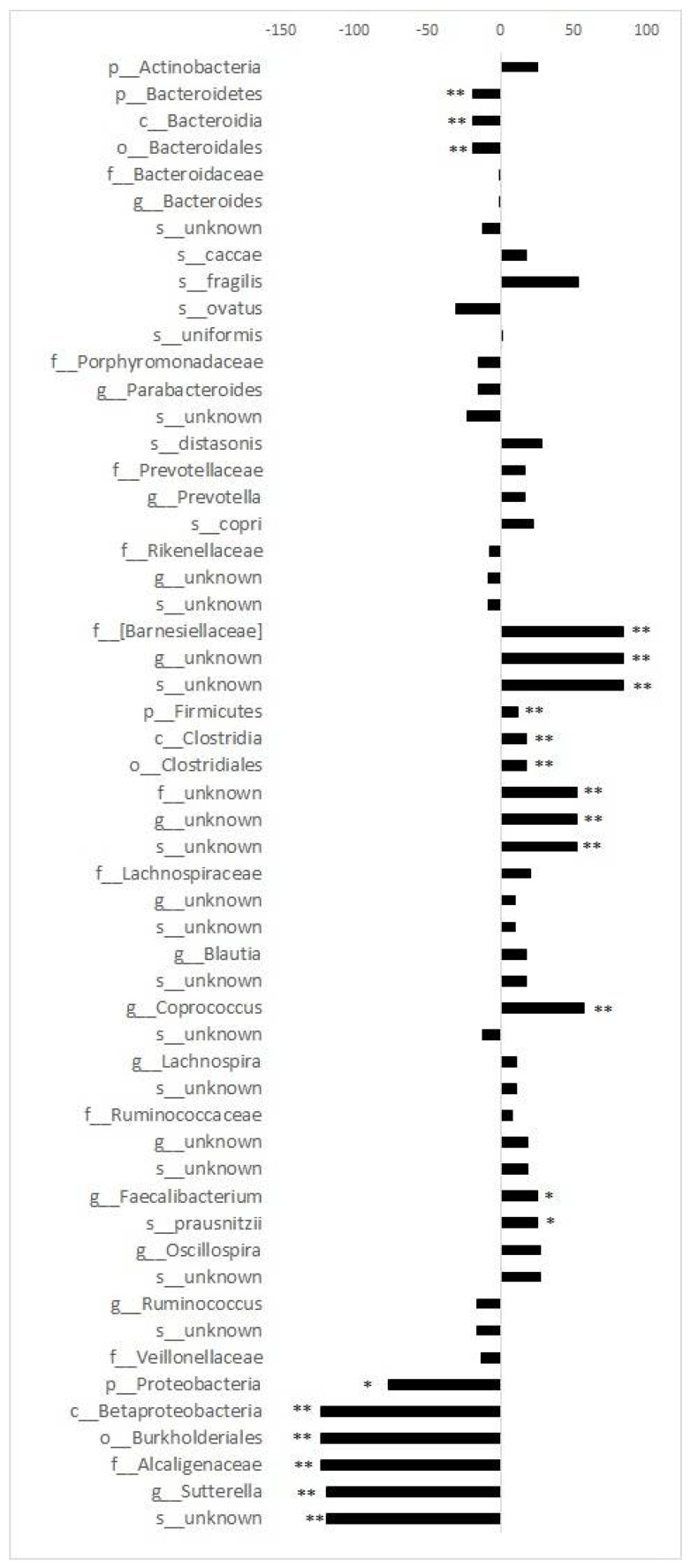
Differences in relative abundance between the highest and the lowest IDQ score quartiles. * FDR < 0.2, ** FDR < 0.1. The *p*-values for the differences were corrected for multiple testing. The false discovery rate (FDR) was 0.2 *, but also those with FDR < 0.1 are shown **. The values represent a median difference as percentage and confidence interval (95% CI).

Dietary quality correlated with the relative abundance of 14 gut bacteria in the various taxonomic levels of the 55 bacteria with relative abundance >1% (Figure 2). Namely, IDQ score correlated directly with genus *Coprococcus* belonging to the family *Lachonspiraceae*, species *Faecalibacterium prausnitzii* in the family *Ruminococcaceae,* and unknown species from the *Barnciellaceae* family, and inversely with an unknown species of genus *Sutterella* belonging to the family *Alcaligenaceae*. In addition, similar associations between IDQ scores and relative abundances were detected between the highest and the lowest IDQ score quartiles, namely, *Coprococcus*, *F. prausnitzii*, and an unknown species in the family *Barnciellaceae* were higher, and an unknown species in the genus *Sutterella* were lower in the top quartile of the IDQ score (Figure 3, Table S1). When investigating the difference in relative abundances between the categorized IDQ, *Coprococcus* (0.75 (0.42–1.46) vs. 1.33 (0.62–2.97), *p* = 0.015) and *Faecalibacterium* (4.71 (2.59–6.47) vs. 5.62 (3.88–7.88), *p* = 0.038) and species *F. prausnitzii* (4.71 (2.59–6.47) vs. 5.62 (3.88–7.88), *p* = 0.038) were higher in women with good dietary quality when compared to those with poor dietary quality, however the differences no longer remained statistically significant when adjusted for multiple testing (adjusted *p* > 0.35) (Table S2).

## 3. Discussion

It was demonstrated here that higher dietary quality was related to a higher gut microbiota diversity. In particular, those individuals adhering best to the dietary recommendations, and those who consumed whole grains and vegetables on a daily basis manifested with the highest gut microbiota diversity. When considering microbiota abundances, the genus *Coprococcus*, *F. prausnitzii,* and an unknown species in family *Barnciellaceae* were related to a higher dietary quality, and an unknown species of genus *Sutterella* were related to lower dietary quality. As both high dietary quality and a high gut microbiota diversity are considered to be characteristics that are likely to yield health benefits, the approach presented here (i.e., the evaluation of dietary intake by a validated quality index), may be useful in the identification of those individuals most in need for dietary counseling.

A similar approach as applied here (i.e., either examining the association between high dietary quality or that of a healthy dietary pattern), and gut microbiota diversity has thus far been rarely investigated and mainly from the perspective of the Mediterranean diet. Similar to our findings, Bowyer and coworkers [19], detected the strongest correlations between the Mediterranean diet score and a healthy eating index with the Shannon diversity index. They also revealed similar correlations with OTUs and the Simpson index. In a small study of 27 adults, a better adherence to the Mediterranean diet as measured by the PREDIMED test was related to a statistically nonsignificant tendency towards a higher Chao index, but no association was seen with either Shannon index or OTUs [20]. Similarly, in an Italian study, no relation was detected between adherence to a Mediterranean dietary pattern with microbiota alpha diversity [21].

As with gut microbiota diversity, the relations between relative abundance of bacteria and dietary scores have been mainly investigated using Mediterranean diet scores [19,20,22], with very variable results. The heterogeneity may be attributable to the many factors related to the methods used in both dietary assessment and analysis of the gut microbiota composition. Compared to our findings, a similar association between *Coprococcus* and dietary quality has been observed in a few studies [19,23]. In the study by Bowyer and coworkers, conducted in twins, *Coprococcus* associated with the Mediterranean diet score, but not with the healthy eating index [19]. An investigation conducted in pregnant women with gestational diabetes, showed that adherence to the dietary recommendations given during pregnancy was related to a higher increase in *Coprococcus,* among some other bacteria [23]. In the same study, a higher abundance of *Facelibacterium* was observed in the adherent women, which is in line with our finding of a higher abundance of *F. prausnitzii*, a bacteria belonging to the genus *Faecalibactrium*. In our previous study, *Barnciellaceae* was positively correlated with a higher intake of dietary fiber, and inversely with dietary fat [24], a result which is in line with the current observations of dietary quality.

*Coprococcus* and *Faecalibacterium*, both belonging to the order Clostridiales, are butyrate, a short chain fatty acid producing bacteria. Short chain fatty acids are considered anti-inflammatory bacterial metabolites [25], but also participate in host energy metabolism. It seems likely that the health benefits related to higher dietary quality may at least partly be explained by the higher abundance of these short chain fatty acid producing bacteria. Little is known about the health influencing properties of *Barnciellaceae* or *Sutterella*. *Barneciellaceae* belongs to the order Bacteroidales, which is highly abundant in the gut, and *Sutterella*, a genus in the order Burkholderiales, also a prevalent commensal in the gut, is postulated to possess pro-inflammatory properties [26]. All in all, more studies are needed to link the properties of the bacteria associated with dietary quality with host health.

In our study the correlation coefficients pointed to a moderate positive association of dietary quality index with microbiota diversity, which was at the same level as previously reported for the Mediterranean diet [20]. The association appears to be of significance, in addition to the dietary intake, other factors such as nutritional status, infections, and medications also define the microbiota composition and diversity. The strength of our study was that we chose to examine a homogenous study population (i.e., overweight and obese pregnant women), and we included in the analysis only women who had not consumed antibiotics within 8 weeks prior to fecal sample collection. We also standardized the sample collection and analytical procedures. The study was conducted in early pregnancy when the physiological need for additional energy is miniscule suggesting that the study results may be generalizable to non-pregnant conditions. Nevertheless, some differences in food intake may be seen already in early pregnancy as compared to the non-pregnant state [27]. Furthermore, in the analysis of the dietary intake, we utilized a validated index, which has been the approach adopted in some [19,20,21], but not in all [23], previous studies. Typically, dietary intake analysis is population specific, and the optimal validation of the dietary index for each group of individuals is needed. Nevertheless, Boyer and co-workers [19] applied two different dietary indexes in their study, and found associations with gut microbiota diversity indexes.

In our study, two components of the dietary quality index, namely whole grains and vegetables, were best related to gut microbiota diversity. Indeed, gut microbes are known to utilize components of the whole grain including fiber and subsequently to produce a range of metabolites, an example being short chain fatty acids with expected health impacts [28]. When one considers vegetable consumption, the composition of the bacterial community has been found to change when cruciferous vegetables were added to a fruit- and vegetable-free diet [29]. A vegetarian diet has also been linked with the presence of fiber-degrading bacteria [21]. Our study is adding to this previous evidence by demonstrating that in order to gain a benefit in gut microbiota diversity, daily consumption of whole grains and vegetables is required, as less frequent consumption was not related to the microbiota diversity.

Whether the same approach as applied in the current study for evaluating the relationship between diet as a whole and dietary index is applicable to other populations and conditions needs to be addressed. For example, the microbiome of the pregnant women may be influenced by stage of pregnancy [23,30,31,32], normal weight, overweight or obesity status [30,33,34,35], and by the presence of pregnancy complications, especially gestational diabetes [1,2]. Furthermore, female hormonal production [36] or gender [37] may contribute to the composition of the microbiome. With respect to the study methods, a metagenomic approach would provide deeper insights into the lower bacterial taxonomies and the function of the microbiome [38]. It is also of note, that the gut microbiota composition varies according to the geographic location, ethnicity, and local environment [39]. There are several specific bacteria that differ according to location, but also differences in diversity have been observed (e.g., an African population had a higher gut microbiota diversity) [12,13].

Based on the results of the current study, the high dietary quality is likely to advance health-promoting gut microbiota diversity. The data may be used in health counseling as the diet was evaluated by a validated low-burden, stand-alone index that does not require another method to analyze food intake for subsequent calculation of index scores. Furthermore, the data may be useful in microbiome studies to control for dietary variance. This is of importance, since even though it is apparent that diet makes an important contribution to the diversity of gut microbiota, the dietary intake is not commonly studied, probably since it is a laborious task for both the study participants and its personnel. The approach introduced in this study allows evaluation of the dietary quality by applying a validated and simple method.

## 4. Materials and Methods

### 4.1. Design and Participants

Dietary quality and fecal microbiota diversity were determined from 84 overweight and obese pregnant women participating in a mother and infant dietary intervention trial (ClinicalTrials.gov, NCT01922791) [40]. The inclusion criteria were self-reported pre-pregnancy BMI ≥ 25 kg/m^2^, less than 18 weeks of gestation, single fetal pregnancy, and absence of chronic metabolic and gastrointestinal diseases including diabetes and inflammatory bowel disease.

The women attended a study visit for the collection of the data on dietary quality and demographics at the baseline of the trial and provision of a fecal sample. For this report only women who had not used antibiotics in the 8 weeks prior to the study visit were included. One fecal sample was collected from the women in sterile plastic pots. Samples were collected the morning of the study visit or the previous evening, delivered to the study unit and kept at +4 °C until DNA extraction.

The study was conducted according to the guidelines laid down in the Declaration of Helsinki, and all procedures involving human subjects were approved by the Ethics Committee of the Hospital District of Southwest Finland (permission code 115/180/2012; approved on 11 December 2012). Written informed consent was obtained from all participants.

### 4.2. Dietary Quality Index

The dietary quality was measured by the validated index of diet quality (IDQ) [9] questionnaire that reflects adherence to dietary recommendations [41]. The IDQ reflects the health-promoting properties of the diet in its entirety. The IDQ needs a short time for completion, has a simple scoring system, and is independent of other dietary assessment methods. The quality of the diet was defined as poor when index points were less than 10 out of the maximum 15 points, and good when points were 10 or more. The questionnaire is composed of 18 questions regarding the frequency and amount of consumption of foods during the preceding week (e.g., whole grains, fats including spreads and salad dressing, fish, dairy, vegetables, fruits and berries, fruit juices, sugar-containing soft drinks, sweets, and chocolate). The criteria for a health-promoting diet were consumption of whole grains (at least 25 g fiber/day, whole-grain bread >100g/day), vegetables, fruits and berries (at least 400g/day), and dairy (>500 g/day) and choice of foods that will yield a good quality of dietary fat intake (saturated fatty acids <10% of energy intake, monounsaturated fatty acids 10%–15% of energy intake, and polyunsaturated fatty acids 5%–10% of energy intake) and low intake of sugar (saccharose <10% of energy intake).

### 4.3. Gut Microbiota Diversity Indexes and Abundancies

The gut microbiota was analyzed from DNA extracted (GTX stool extraction kit and fully automated GenoXTract machine (Hain Lifescience, Nehren, Germany)) from fecal samples as described earlier [42,43]. The DNA samples were sequenced in Sequencing and Bioinformatics Service at Fundación para el Fomento de la Investigación Sanitaria y Biomédica de la Comunitat Valenciana (FISABIO) (Valencia, Spain). The 16S ribosomal amplicons were amplified following the 16S Metagenomic Sequencing Library Preparation Illumina protocol (Part # 15044223 Rev. A). The gene-specific sequences used in this protocol targeted the 16S V3 and V4 region. Illumina adapter overhang nucleotide sequences were added to the gene-specific sequences. The primers were selected from Klindworth et al. 2013 [44].

The gut microbiota analysis provided 41,000 to 118,000 sequences/sample (approximately 25% having >100,000 reads), the amount which is in accordance to what is typically obtained using MiSeq for statistical analysis. Raw sequences were processed using the QIIME software package (v.1.9, http://qiime.org/)) [45]. The data were sub-sampled to 41,000 reads to normalize the samples. OTUs were chosen at 97% similarity against the Greengenes database and matched with known bacterial genomes to identify members of the fecal community. Four measures for the gut microbiota diversity and richness were used: Chao (species based index), observed OTUs, PD (phylogenetic differences among species), and Shannon index (diversity index) [46]. The diversity and richness estimators were calculated at an alpha rarefaction sequence depth of 36382.0. The abundance of bacteria >1% of total microbiota was considered to be reliable and taken for further analyses.

### 4.4. Statistics

The data was evaluated for normality by using the Kolmogorov–Smirnov test and by visual inspection of the histograms. Since not all variables were normally distributed, the Mann–Whitney *U* test was used to compare the differences in gut microbiota diversity between the poor and good dietary quality, between the lowest and the highest quartile of dietary quality index, and between the daily consumption (seven days/week compared to six or less days per week) of whole grains and vegetables. Similarly, the Mann–Whitney *U* test was performed to analyze the relative abundance of the bacteria between poor and good dietary quality and between the lowest and highest quartiles of dietary quality score. Spearman’s correlation was utilized for correlations between dietary quality index and individual IDQ food components with gut microbiota diversity or relative abundance of the bacteria. The *p*-values for the relative abundances were corrected for multiple testing, using 0.2 for the false discovery rate (FDR). The results are shown as median and interquartile range (IQR) or median difference as percentage and confidence interval (95% CI). Baseline characteristics are shown as mean (SD) or median (IQR). Statistical analyses were performed with SPSS for Windows, version 24 (IBM Corp., Armonk, NY, USA).

## Figures and Tables

**Table 1:** Participating women (*n* = 84) ^1^.

Variable	Values
**Characteristics**	
Age (years)	30.1 (4.7)
Pre-pregnancy BMI (kg/m^2^)	30.3 (4.6)
Obese	40/84 (48%)
Overweight	44/84 (52%)
Primipara	31% (26/84)
University degree	58% (45/77)
Gestational weeks	14.0 (11.0–15.0)
**Dietary Quality**	
IDQ score	9.84 (8.0–11.97)
Good dietary quality (IDQ scores ≥10)	49% (41/84)
**Gut microbiota Diversity Index**	
PD	36.9 (31.3–39.9)
Chao	390.1 (346.0–421.3)
Observed OTUs	343.7 (296.4–373.8)
Shannon	5.4 (5.2–5.8)

^1^ Data are presented as mean (SD) or median and interquartile range (IQR) or as *n*/out of *total n* (%).

**Table 2:** Gut microbiota diversity indexes in the lowest and the highest diet quality score (IDQ) quartile ^1^.

Index	IDQ Lowest Quartile (*n* = 25) 7 (5.9–7.5)	IDQ Highest Quartile (*n* = 21) 13 (12.0–13.0)	*p*-Value ^2^
Shannon	5.3 (4.8–5.5)	5.7 (5.4–6.0)	0.001
PD	35.4 (29.7–38.8)	39.7 (35.3–41.5)	0.010
Chao	374.5 (326.3–403.3)	415.0 (380.3–423.9)	0.019
Observed OTUs	333.7 (284.5–358.9)	363.2 (334.7–374.0)	0.032

^1^ Data are presented as median (IQR). ^2^ Mann–Whitney U test between the IDQ score quartiles.

**Table 3:** Gut microbiota diversity indexes in the women with good (IDQ ≥10) and poor (IDQ <10) dietary quality ^1^.

Index	IDQ < 10, (*n* = 43)	IDQ ≥ 10, (*n* = 41)	*p*-Value ^2^
Shannon	5.4 (4.9–5.6)	5.7 (5.4–5.9)	0.004
PD	34.7 (30.0–39.0)	39.4 (33.3–41.2)	0.055
Chao	383.9 (342.6–419.9)	407.5 (352.2–431.5)	0.087
Observed OTUs	330.1 (292.9–367.4)	360.8 (313.8–380.5)	0.052

^1^ Data are presented as median (IQR). ^2^ Mann–Whitney U test between the women in the poor and the good dietary quality classes.

**Table 4:** Difference in Shannon index according to frequency of food consumption ^1^.

IDQ Food Group	Consumption Frequency		*p*-Value ^2^
	7 Days/Week	≤6 Days/Week	
Whole grains	5.6 (5.3–5.9)	5.4 (4.9–5.7)	0.031
Vegetables	5.6 (5.3–5.9)	5.4 (4.9–5.6)	0.016

^1^ Data are presented as median (IQR). ^2^ Mann–Whitney U test between the IDQ food groups.

## Supplementary Materials

Supplementary materials can be found at https://www.mdpi.com/1422-0067/20/8/1835/s1. Table S1. Difference in relative abundance of bacteria between the individuals in the lowest and the highest quartile of diet quality score (IDQ). Table S2. Difference in relative abundance of bacteria between the individuals in the poor and good dietary quality classes (categorized IDQ).

## Abbreviations

IDQ	Index of diet quality
OTU	Operational taxonomic unit
PD	Phylogenetic diversity
