# Supplementary material for: Overall Dietary Quality Relates to Gut Microbiota Diversity and Abundance
Source: Int J Mol Sci. 2019 Apr 13;20(8):1835. doi: 10.3390/ijms20081835 (PMC6515207; doi:10.3390/ijms20081835)
Supplement: Supplementary file 1 [file ijms-20-01835-s001.pdf]

Table S1

	IDQ Q1, n=25			IDQ Q4, n=21								
	median	IQR 25 percentiles	IQR 75 percentiles	median	IQR 25 percentiles	IQR 75 percentiles	% difference between median of obese and overweight women	median difference	95%CI lower	95%CI higher	P-value, Mann Whitney U test	BH-adjusted P-value
k__Bacteria;p__Actinobacteria	0,65	0,31	1,13	0,86	0,37	2,26	25,2	0,22	-0,009	0,001	0,16	0,39
k__Bacteria;p__Bacteroidetes	52,23	45,03	64,65	44,01	38,09	56,03	-18,7	-8,22	0,006	0,148	0,03	0,09
k__Bacteria;p__Firmicutes	42,22	33,07	49,91	47,97	39,09	56,60	12,0	5,75	-0,157	-0,007	0,03	0,09
k__Bacteria;p__Proteobacteria	2,96	1,60	4,62	1,68	0,82	3,31	-76,6	-1,28	0	0,021	0,07	0,18
k__Bacteria;p__Bacteroidetes;c__Bacteroidia	52,23	45,03	64,65	44,01	38,09	56,03	-18,7	-8,22	0,006	0,148	0,03	0,09
k__Bacteria;p__Firmicutes;c__Clostridia	39,35	32,51	49,30	47,52	38,39	55,62	17,2	8,17	-0,15	-0,005	0,03	0,09
k__Bacteria;p__Proteobacteria;c__Betaproteobacteria	1,64	0,75	3,51	0,74	0,22	1,43	-122,6	-0,91	0,002	0,017	0,01	0,07
k__Bacteria;p__Bacteroidetes;c__Bacteroidia;o__Bacteroidales	52,23	45,03	64,65	44,01	38,09	56,03	-18,7	-8,22	0,006	0,148	0,03	0,09
k__Bacteria;p__Firmicutes;c__Clostridia;o__Clostridiales	39,22	32,51	49,29	47,47	38,36	55,57	17,4	8,24	-0,015	-0,006	0,03	0,09
k__Bacteria;p__Proteobacteria;c__Betaproteobacteria;o__Burkholderiales	1,64	0,75	3,51	0,74	0,22	1,43	-122,6	-0,91	0,002	0,017	0,01	0,07
k__Bacteria;p__Bacteroidetes;c__Bacteroidia;o__Bacteroidales;f__Bacteroidaceae	29,92	24,19	43,48	29,82	18,39	37,29	-0,3	-0,10	-0,049	0,107	0,28	0,53
k__Bacteria;p__Bacteroidetes;c__Bacteroidia;o__Bacteroidales;f__Porphyromonadaceae	3,83	2,21	5,17	3,32	2,20	5,56	-15,4	-0,51	-0,013	0,014	0,85	0,92
k__Bacteria;p__Bacteroidetes;c__Bacteroidia;o__Bacteroidales;f__Prevotellaceae	0,00	0,00	7,68	0,01	0,00	0,01	16,6	0,00	0	0	0,51	0,66
k__Bacteria;p__Bacteroidetes;c__Bacteroidia;o__Bacteroidales;f__Rikenellaceae	4,85	2,69	9,92	4,53	3,53	6,37	-7,1	-0,32	-0,013	0,032	0,53	0,66
k__Bacteria;p__Bacteroidetes;c__Bacteroidia;o__Bacteroidales;f__[Barnesiellaceae]	0,43	0,00	2,14	2,60	1,58	4,03	83,7	2,18	-0,026	-0,003	0,01	0,07
k__Bacteria;p__Firmicutes;c__Clostridia;o__Clostridiales;f__	2,87	1,70	4,47	6,06	2,64	10,16	52,7	3,19	-0,046	0	0,02	0,09
k__Bacteria;p__Firmicutes;c__Clostridia;o__Clostridiales;f__Lachnospiraceae	13,98	11,04	18,67	17,51	12,34	20,48	20,2	3,53	-0,049	0,014	0,32	0,53
k__Bacteria;p__Firmicutes;c__Clostridia;o__Clostridiales;f__Ruminococcaceae	18,51	10,56	21,69	20,05	15,21	27,79	7,7	1,54	-0,087	0,013	0,18	0,40
k__Bacteria;p__Firmicutes;c__Clostridia;o__Clostridiales;f__Veillonellaceae	1,01	0,62	2,13	0,89	0,55	1,44	-13,7	-0,12	-0,003	0,007	0,52	0,66
k__Bacteria;p__Proteobacteria;c__Betaproteobacteria;o__Burkholderiales;f__Alcaligenaceae	1,64	0,75	3,51	0,74	0,22	1,43	-122,6	-0,91	0,002	0,017	0,01	0,07
k__Bacteria;p__Bacteroidetes;c__Bacteroidia;o__Bacteroidales;f__Bacteroidaceae;g__Bacteroides	29,92	24,19	43,48	29,82	18,39	37,29	-0,3	-0,10	-0,049	0,107	0,28	0,53
k__Bacteria;p__Bacteroidetes;c__Bacteroidia;o__Bacteroidales;f__Porphyromonadaceae;g__Parabacteroides	3,83	2,21	5,17	3,31	2,20	5,37	-15,5	-0,51	-0,012	0,015	0,77	0,90
k__Bacteria;p__Bacteroidetes;c__Bacteroidia;o__Bacteroidales;f__Prevotellaceae;g__Prevotella	0,00	0,00	7,68	0,01	0,00	0,01	16,6	0,00	0	0	0,51	0,66
k__Bacteria;p__Bacteroidetes;c__Bacteroidia;o__Bacteroidales;f__Rikenellaceae;g__	4,84	2,69	9,91	4,45	3,53	6,37	-8,6	-0,38	-0,013	0,033	0,52	0,66
k__Bacteria;p__Bacteroidetes;c__Bacteroidia;o__Bacteroidales;f__[Barnesiellaceae];g__	0,43	0,00	2,14	2,60	1,58	4,03	83,7	2,18	-0,025	-0,003	0,01	0,07
k__Bacteria;p__Firmicutes;c__Clostridia;o__Clostridiales;f__;g__	2,87	1,70	4,47	6,06	2,64	10,16	52,7	3,19	-0,046	0	0,02	0,09
k__Bacteria;p__Firmicutes;c__Clostridia;o__Clostridiales;f__Lachnospiraceae;g__	7,34	5,51	11,67	8,12	5,62	10,47	9,6	0,78	-0,021	0,027	0,92	0,92
k__Bacteria;p__Firmicutes;c__Clostridia;o__Clostridiales;f__Lachnospiraceae;g__Blautia	1,49	1,27	2,09	1,81	1,06	2,76	17,6	0,32	-0,01	0,003	0,47	0,66
k__Bacteria;p__Firmicutes;c__Clostridia;o__Clostridiales;f__Lachnospiraceae;g__Coprococcus	0,75	0,36	1,25	1,76	0,64	3,63	57,3	1,01	-0,018	-0,002	0,01	0,09
k__Bacteria;p__Firmicutes;c__Clostridia;o__Clostridiales;f__Lachnospiraceae;g__Lachnospira	1,24	0,52	3,16	1,39	0,63	3,19	10,7	0,15	-0,01	0,007	0,73	0,88
k__Bacteria;p__Firmicutes;c__Clostridia;o__Clostridiales;f__Ruminococcaceae;g__	7,79	2,40	11,26	9,53	6,77	14,21	18,3	1,74	-0,061	0,013	0,32	0,53
k__Bacteria;p__Firmicutes;c__Clostridia;o__Clostridiales;f__Ruminococcaceae;g__Faecalibacterium	4,18	2,50	6,05	5,62	3,66	8,26	25,6	1,44	-0,032	0,003	0,07	0,18
k__Bacteria;p__Firmicutes;c__Clostridia;o__Clostridiales;f__Ruminococcaceae;g__Oscillospira	0,90	0,53	1,72	1,24	0,89	1,59	27,4	0,34	-0,006	0,002	0,32	0,53
k__Bacteria;p__Firmicutes;c__Clostridia;o__Clostridiales;f__Ruminococcaceae;g__Ruminococcus	2,85	0,96	4,45	2,46	0,80	5,11	-15,7	-0,39	-0,016	0,012	0,90	0,92
k__Bacteria;p__Proteobacteria;c__Betaproteobacteria;o__Burkholderiales;f__Alcaligenaceae;g__Sutterella	1,33	0,70	3,51	0,61	0,19	1,40	-118,6	-0,72	0,002	0,018	0,01	0,07
k__Bacteria;p__Bacteroidetes;c__Bacteroidia;o__Bacteroidales;f__Bacteroidaceae;g__Bacteroides;s__	19,22	13,25	31,78	17,14	11,87	24,66	-12,1	-2,08	-0,038	0,104	0,41	0,62
k__Bacteria;p__Bacteroidetes;c__Bacteroidia;o__Bacteroidales;f__Bacteroidaceae;g__Bacteroides;s__caccae	0,82	0,09	1,90	1,00	0,36	2,78	17,5	0,17	-0,01	0,003	0,37	0,60
k__Bacteria;p__Bacteroidetes;c__Bacteroidia;o__Bacteroidales;f__Bacteroidaceae;g__Bacteroides;s__fragilis	0,27	0,00	0,85	0,57	0,02	1,71	53,0	0,30	-0,006	0,001	0,27	0,53
k__Bacteria;p__Bacteroidetes;c__Bacteroidia;o__Bacteroidales;f__Bacteroidaceae;g__Bacteroides;s__ovatus	1,43	0,77	2,49	1,10	0,72	2,77	-30,1	-0,33	-0,008	0,007	0,87	0,92
k__Bacteria;p__Bacteroidetes;c__Bacteroidia;o__Bacteroidales;f__Bacteroidaceae;g__Bacteroides;s__uniformis	3,72	1,21	7,85	3,74	1,67	7,48	0,6	0,02	-0,022	0,02	0,83	0,92
k__Bacteria;p__Bacteroidetes;c__Bacteroidia;o__Bacteroidales;f__Porphyromonadaceae;g__Parabacteroides;s__	2,28	0,50	4,66	1,85	0,69	3,82	-23,3	-0,43	-0,008	0,019	0,40	0,62
k__Bacteria;p__Bacteroidetes;c__Bacteroidia;o__Bacteroidales;f__Porphyromonadaceae;g__Parabacteroides;s__distasonis	0,90	0,00	1,86	1,25	0,46	2,56	28,0	0,35	-0,012	0,002	0,15	0,37
k__Bacteria;p__Bacteroidetes;c__Bacteroidia;o__Bacteroidales;f__Prevotellaceae;g__Prevotella;s__copri	0,00	0,00	0,01	0,01	0,00	0,01	22,7	0,00	0	0	0,28	0,53
k__Bacteria;p__Bacteroidetes;c__Bacteroidia;o__Bacteroidales;f__Rikenellaceae;g__;s__	4,84	2,69	9,91	4,45	3,53	6,37	-8,6	-0,38	-0,013	0,033	0,52	0,66
k__Bacteria;p__Bacteroidetes;c__Bacteroidia;o__Bacteroidales;f__[Barnesiellaceae];g__;s__	0,43	0,00	2,14	2,60	1,58	4,03	83,7	2,18	-0,026	-0,003	0,01	0,07
k__Bacteria;p__Firmicutes;c__Clostridia;o__Clostridiales;f__;g__;s__	2,87	1,70	4,47	6,06	2,64	10,16	52,7	3,19	-0,046	0	0,02	0,09
k__Bacteria;p__Firmicutes;c__Clostridia;o__Clostridiales;f__Lachnospiraceae;g__;s__	7,34	5,51	11,67	8,12	5,62	10,47	9,6	0,78	-0,021	0,027	0,92	0,92
k__Bacteria;p__Firmicutes;c__Clostridia;o__Clostridiales;f__Lachnospiraceae;g__Blautia;s__	1,49	1,27	2,09	1,81	1,06	2,76	17,7	0,32	-0,01	0,003	0,50	0,66
k__Bacteria;p__Firmicutes;c__Clostridia;o__Clostridiales;f__Lachnospiraceae;g__Coprococcus;s__	0,69	0,29	1,18	0,61	0,39	1,43	-12,7	-0,08	-0,005	0,002	0,82	0,92
k__Bacteria;p__Firmicutes;c__Clostridia;o__Clostridiales;f__Lachnospiraceae;g__Lachnospira;s__	1,24	0,52	3,16	1,39	0,63	3,19	10,7	0,15	-0,01	0,007	0,73	0,88
k__Bacteria;p__Firmicutes;c__Clostridia;o__Clostridiales;f__Ruminococcaceae;g__;s__	7,79	2,40	11,26	9,53	6,77	14,21	18,3	1,74	-0,061	0,013	0,32	0,53
k__Bacteria;p__Firmicutes;c__Clostridia;o__Clostridiales;f__Ruminococcaceae;g__Faecalibacterium;s__prausnitzii	4,18	2,50	6,05	5,62	3,66	8,26	25,6	1,44	-0,032	0,003	0,07	0,18
k__Bacteria;p__Firmicutes;c__Clostridia;o__Clostridiales;f__Ruminococcaceae;g__Oscillospira;s__	0,90	0,53	1,72	1,24	0,89	1,59	27,4	0,34	-0,006	0,002	0,32	0,53
k__Bacteria;p__Firmicutes;c__Clostridia;o__Clostridiales;f__Ruminococcaceae;g__Ruminococcus;s__	2,85	0,96	4,45	2,46	0,80	5,11	-15,7	-0,39	-0,016	0,012	0,90	0,92
k__Bacteria;p__Proteobacteria;c__Betaproteobacteria;o__Burkholderiales;f__Alcaligenaceae;g__Sutterella;s__	1,33	0,70	3,51	0,61	0,19	1,40	-118,6	-0,72	0,002	0,018	0,01	0,07

Table S2

	poor dietary quality, n=43			good dietary quality, n=41				
	median	IQR 25 percentiles	IQR 75 percentiles	median	IQR 25 percentiles	IQR 75 percentiles	P-value Mann Whitney U test	BH-adjusted P-value
k__Bacteria;p__Actinobacteria	0,65	0,34	1,58	0,79	0,35	1,71	0,582	0,73
k__Bacteria;p__Bacteroidetes	50,76	44,72	59,07	48,44	39,86	56,06	0,167	0,44
k__Bacteria;p__Firmicutes	42,99	34,30	48,86	46,25	38,16	54,21	0,116	0,35
k__Bacteria;p__Proteobacteria	2,96	1,23	4,43	2,22	1,34	3,71	0,205	0,45
k__Bacteria;p__Bacteroidetes;c__Bacteroidia	50,76	44,72	59,07	48,44	39,86	56,06	0,167	0,44
k__Bacteria;p__Firmicutes;c__Clostridia	42,64	33,76	48,02	45,84	37,53	53,55	0,093	0,35
k__Bacteria;p__Proteobacteria;c__Betaproteobacteria	1,64	0,79	2,81	1,09	0,56	1,89	0,11	0,35
k__Bacteria;p__Bacteroidetes;c__Bacteroidia;o__Bacteroidales	50,76	44,72	59,07	48,44	39,86	56,06	0,167	0,44
k__Bacteria;p__Firmicutes;c__Clostridia;o__Clostridiales	42,57	33,76	47,99	45,64	37,50	53,46	0,09	0,35
k__Bacteria;p__Proteobacteria;c__Betaproteobacteria;o__Burkholderiales	1,64	0,79	2,81	1,09	0,56	1,89	0,11	0,35
k__Bacteria;p__Bacteroidetes;c__Bacteroidia;o__Bacteroidales;f__Bacteroidaceae	33,01	24,27	42,56	29,82	20,24	39,92	0,19	0,44
k__Bacteria;p__Bacteroidetes;c__Bacteroidia;o__Bacteroidales;f__Porphyromonadaceae	2,87	1,94	4,69	3,42	2,37	5,37	0,327	0,56
k__Bacteria;p__Bacteroidetes;c__Bacteroidia;o__Bacteroidales;f__Prevotellaceae	0,00	0,00	0,01	0,01	0,00	0,01	0,422	0,60
k__Bacteria;p__Bacteroidetes;c__Bacteroidia;o__Bacteroidales;f__Rikenellaceae	6,32	2,92	9,28	5,36	3,67	8,70	0,876	0,94
k__Bacteria;p__Bacteroidetes;c__Bacteroidia;o__Bacteroidales;f__[Barnesiellaceae]	0,47	0,00	2,48	1,93	0,01	3,03	0,094	0,35
k__Bacteria;p__Firmicutes;c__Clostridia;o__Clostridiales;f__	3,25	1,90	5,22	4,21	2,09	7,10	0,285	0,52
k__Bacteria;p__Firmicutes;c__Clostridia;o__Clostridiales;f__Lachnospiraceae	15,45	11,09	19,45	16,95	13,04	20,78	0,558	0,72
k__Bacteria;p__Firmicutes;c__Clostridia;o__Clostridiales;f__Ruminococcaceae	18,51	13,11	22,37	20,71	15,21	26,53	0,102	0,35
k__Bacteria;p__Firmicutes;c__Clostridia;o__Clostridiales;f__Veillonellaceae	1,09	0,59	2,20	1,16	0,56	2,46	0,869	0,94
k__Bacteria;p__Proteobacteria;c__Betaproteobacteria;o__Burkholderiales;f__Alcaligenaceae	1,64	0,79	2,81	1,09	0,56	1,89	0,11	0,35
k__Bacteria;p__Bacteroidetes;c__Bacteroidia;o__Bacteroidales;f__Bacteroidaceae;g__Bacteroides	33,01	24,27	42,56	29,82	20,24	39,92	0,19	0,44
k__Bacteria;p__Bacteroidetes;c__Bacteroidia;o__Bacteroidales;f__Porphyromonadaceae;g__Parabacteroides	2,87	1,93	4,69	3,42	2,36	5,23	0,35	0,56
k__Bacteria;p__Bacteroidetes;c__Bacteroidia;o__Bacteroidales;f__Prevotellaceae;g__Prevotella	0,00	0,00	0,01	0,01	0,00	0,01	0,422	0,60
k__Bacteria;p__Bacteroidetes;c__Bacteroidia;o__Bacteroidales;f__Rikenellaceae;g__	6,32	2,92	9,25	5,36	3,67	8,70	0,869	0,94
k__Bacteria;p__Bacteroidetes;c__Bacteroidia;o__Bacteroidales;f__[Barnesiellaceae];g__	0,47	0,00	2,48	1,93	0,01	3,03	0,094	0,35
k__Bacteria;p__Firmicutes;c__Clostridia;o__Clostridiales;f__;g__	3,25	1,90	5,22	4,21	2,09	7,10	0,285	0,52
k__Bacteria;p__Firmicutes;c__Clostridia;o__Clostridiales;f__Lachnospiraceae;g__	8,57	5,52	12,18	7,89	5,54	10,69	0,564	0,72
k__Bacteria;p__Firmicutes;c__Clostridia;o__Clostridiales;f__Lachnospiraceae;g__Blautia	1,62	1,25	2,26	1,75	0,94	2,58	0,778	0,93
k__Bacteria;p__Firmicutes;c__Clostridia;o__Clostridiales;f__Lachnospiraceae;g__Coprococcus	0,75	0,42	1,46	1,33	0,62	2,97	0,015	0,35
k__Bacteria;p__Firmicutes;c__Clostridia;o__Clostridiales;f__Lachnospiraceae;g__Lachnospira	1,24	0,53	2,72	1,69	0,63	3,27	0,345	0,56
k__Bacteria;p__Firmicutes;c__Clostridia;o__Clostridiales;f__Ruminococcaceae;g__	8,27	2,68	12,50	9,53	6,55	14,21	0,222	0,45
k__Bacteria;p__Firmicutes;c__Clostridia;o__Clostridiales;f__Ruminococcaceae;g__Faecalibacterium	4,71	2,59	6,47	5,62	3,88	7,88	0,038	0,35
k__Bacteria;p__Firmicutes;c__Clostridia;o__Clostridiales;f__Ruminococcaceae;g__Oscillospira	0,91	0,73	1,45	1,24	0,89	1,52	0,083	0,35
k__Bacteria;p__Firmicutes;c__Clostridia;o__Clostridiales;f__Ruminococcaceae;g__Ruminococcus	2,85	0,98	4,89	2,43	0,74	3,92	0,413	0,60
k__Bacteria;p__Proteobacteria;c__Betaproteobacteria;o__Burkholderiales;f__Alcaligenaceae;g__Sutterella	1,37	0,71	2,81	1,02	0,51	1,89	0,106	0,35
k__Bacteria;p__Bacteroidetes;c__Bacteroidia;o__Bacteroidales;f__Bacteroidaceae;g__Bacteroides;s__	20,30	12,67	31,49	18,25	13,47	26,17	0,359	0,56
k__Bacteria;p__Bacteroidetes;c__Bacteroidia;o__Bacteroidales;f__Bacteroidaceae;g__Bacteroides;s__caccae	0,83	0,10	1,77	0,93	0,34	2,27	0,466	0,64
k__Bacteria;p__Bacteroidetes;c__Bacteroidia;o__Bacteroidales;f__Bacteroidaceae;g__Bacteroides;s__fragilis	0,47	0,01	1,42	0,42	0,03	1,10	0,993	0,99
k__Bacteria;p__Bacteroidetes;c__Bacteroidia;o__Bacteroidales;f__Bacteroidaceae;g__Bacteroides;s__ovatus	1,50	0,79	2,59	1,51	0,82	3,08	0,897	0,95
k__Bacteria;p__Bacteroidetes;c__Bacteroidia;o__Bacteroidales;f__Bacteroidaceae;g__Bacteroides;s__uniformis	3,72	1,53	8,34	3,39	1,89	7,61	0,982	0,99
k__Bacteria;p__Bacteroidetes;c__Bacteroidia;o__Bacteroidales;f__Porphyromonadaceae;g__Parabacteroides;s__	1,48	0,05	3,70	1,86	0,69	3,82	0,989	0,99
k__Bacteria;p__Bacteroidetes;c__Bacteroidia;o__Bacteroidales;f__Porphyromonadaceae;g__Parabacteroides;s__distasonis	1,12	0,00	1,73	1,22	0,55	2,22	0,188	0,44
k__Bacteria;p__Bacteroidetes;c__Bacteroidia;o__Bacteroidales;f__Prevotellaceae;g__Prevotella;s__copri	0,00	0,00	0,01	0,01	0,00	0,01	0,079	0,35
k__Bacteria;p__Bacteroidetes;c__Bacteroidia;o__Bacteroidales;f__Rikenellaceae;g__;s__	6,32	2,92	9,25	5,36	3,67	8,70	0,869	0,94
k__Bacteria;p__Bacteroidetes;c__Bacteroidia;o__Bacteroidales;f__[Barnesiellaceae];g__;s__	0,47	0,00	2,48	1,93	0,01	3,03	0,094	0,35
k__Bacteria;p__Firmicutes;c__Clostridia;o__Clostridiales;f__;g__;s__	3,25	1,90	5,22	4,21	2,09	7,10	0,285	0,52
k__Bacteria;p__Firmicutes;c__Clostridia;o__Clostridiales;f__Lachnospiraceae;g__;s__	8,57	5,52	12,18	7,89	5,54	10,69	0,564	0,72
k__Bacteria;p__Firmicutes;c__Clostridia;o__Clostridiales;f__Lachnospiraceae;g__Blautia;s__	1,61	1,25	2,25	1,75	0,93	2,58	0,792	0,93
k__Bacteria;p__Firmicutes;c__Clostridia;o__Clostridiales;f__Lachnospiraceae;g__Coprococcus;s__	0,69	0,39	1,42	0,61	0,39	1,08	0,651	0,80
k__Bacteria;p__Firmicutes;c__Clostridia;o__Clostridiales;f__Lachnospiraceae;g__Lachnospira;s__	1,24	0,53	2,72	1,69	0,63	3,27	0,345	0,56
k__Bacteria;p__Firmicutes;c__Clostridia;o__Clostridiales;f__Ruminococcaceae;g__;s__	8,27	2,68	12,50	9,53	6,55	14,21	0,222	0,45
k__Bacteria;p__Firmicutes;c__Clostridia;o__Clostridiales;f__Ruminococcaceae;g__Faecalibacterium;s__prausnitzii	4,71	2,59	6,47	5,62	3,88	7,88	0,038	0,35
k__Bacteria;p__Firmicutes;c__Clostridia;o__Clostridiales;f__Ruminococcaceae;g__Oscillospira;s__	0,91	0,73	1,45	1,24	0,89	1,52	0,083	0,35
k__Bacteria;p__Firmicutes;c__Clostridia;o__Clostridiales;f__Ruminococcaceae;g__Ruminococcus;s__	2,85	0,98	4,89	2,43	0,74	3,92	0,413	0,60
k__Bacteria;p__Proteobacteria;c__Betaproteobacteria;o__Burkholderiales;f__Alcaligenaceae;g__Sutterella;s__	1,37	0,71	2,81	1,02	0,51	1,89	0,106	0,35
